# The Biological Functions and Clinical Significance of SARS-CoV-2 Variants of Corcern

**DOI:** 10.3389/fmed.2022.849217

**Published:** 2022-05-20

**Authors:** Hikmet Akkız

**Affiliations:** Department of Gastroenterology and Hepatology, The University of Çukurova, Adana, Turkey

**Keywords:** SARS-CoV-2, COVID-19, variant of concern, the Alpha variant, the Beta variant, the Gamma variant, the Delta variant, the Omicron variant

## Abstract

Severe acute respiratory syndrome coronavirus 2 (SARS-CoV-2) is continuing to evolve, emerging novel variants with spike protein mutations. Although most mutations emerged in the SARS-CoV-2 genome are neutral or mildly deleterious, a small number of mutations can affect virus phenotype that confers the virus a fitness advantage. These mutations can enhance viral replication, raise the risk of reinfection and blunt the potency of neutralizing antibodies triggered by previous infection and vaccination. Since December 2020, the SARS-CoV-2 has emerged five quickly spreading strains, designated variants of concern (VOCs), including the Alpha (B.1.1.7) variant, the Beta (B.1.351) variant, the Gamma (P.1) variant, the Delta (B.1.617.2) variant and the Omicron (B.1.1.529) variant. These variants have a high number of the mutations in the spike protein that promotes viral cell entry through the angiotensin-converting enzyme -2 (ACE2). Mutations that have arisen in the receptor binding domain (RBD) of the spike protein are of great concern due to their potential to evade neutralizing antibodies triggered by previous infection and vaccines. The Alpha variant emerged in the United Kingdom in the second half of 2020 that has spread quickly globally and acquired the E484K mutation in the United Kingdom and the United States. The Beta and Gamma variants emerged in South Africa and Brazil, respectively, that have additional mutations at positions E484 and K417 in the RBD. SARS-CoV-2 variants containing the combination of N501Y, E484K, and K417N/T mutations exhibit remarkably decreased sensitivity to neutralizing antibodies mediated by vaccination or previous infection. The Gamma variant may result in more severe disease than other variants do even in convalescent individuals. The Delta variant emerged in India in December 2020 and has spread to many countries including the United States and the United Kingdom. The Delta variant has 8 mutations in the spike protein, some of which can influence immune responses to the key antigenic regions of RBD. In early November 2021, the Omicron (B.1.1.529) variant was first detected in Botswana and South Africa. The Omicron variant harbors more than 30 mutations in the spike protein, many of which are located within the RBD, which have been associated with increased transmissibility and immune evasion after previous infection and vaccination. Additionally, the Omicron variant contains 3 deletions and one insertion in the spike protein. Recently, the Omicron variant has been classified into three sublineages, including BA.1, BA.2, and BA.3, with strikingly different genetic characteristics. The Omicron BA.2 sublineage has different virological landscapes, such as transmissibility, pathogenicity and resistance to the vaccine-induced immunity compared to BA.1 and BA.3 sublineages. Mutations emerged in the RBD of the spike protein of VOCs increase viral replication, making the virus more infectious and more transmissible and enable the virus to evade vaccine-elicited neutralizing antibodies. Unfortunately, the emergence of novel SARS-CoV-2 VOCs has tempered early optimism regarding the efficacy of COVID-19 vaccines. This review addresses the biological and clinical significance of SARS-CoV-2 VOCs and their impact on neutralizing antibodies mediated by existing COVID-19 vaccines.

## Introduction

Three coronaviruses have caused life-threating severe diseases in humans during the last two decades: severe acute respiratory syndrome coronavirus (SARS-CoV), Middle-East respiratory syndrome coronavirus (MERS-CoV) and severe acute respiratory syndrome coronavirus 2 (SARS-CoV-2) (1–3). SARS-CoV emerged in China, in 2002 and caused a global pandemic in 2003 with an approximately 10% case fatality rate (CFR) (1). MERS-CoV was first reported in Saudi Arabia in 2012, where it continues a major public health problem, and has spread to many countries (2). SARS-CoV-2 has been detected in December 2019 in Wuhan, Hubei province of China and has spread quickly worldwide resulting in over million recorded patients of COVID-19 and over million deaths (3, 4). The SARS-CoV-2 is an envelope, positive-sense, single-stranded RNA virus which belongs to the betacoronaviridae family (5–7). The sequencing studies of three recently detected coronaviruses documented that SARS-CoV-2 exhibits 79 and 50% sequence similarity with SARS-CoV and MERS-CoV, respectively (6). Recognition of the receptor is the initial step of viral infection and is a key determinant of host cell and tissue tropism (5, 8, 9). The binding affinity of the spike glycoprotein to the angiotensin-converting enzyme 2 (ACE2) receptor influences the SARS-CoV-2 replication fitness and disease severity in humans (5, 8, 10). The spike protein is a homotrimeric class I fusion glycoprotein that contains two functionally different parts, including S1 and S2 subunits (5, 6, 8, 11). The S1 subunit comprises the receptor-binding domain (RBD) that engaged the host cell receptor which may determine virus and host cell tropism (5, 8, 9). The RBD is the key player within the S1 subunit that contains a core structure and receptor binding motif (RBM), which is the most variable part of spike protein (5, 6, 10, 12). Transmembrane S2 subunit includes heptad repead regions and the fusion peptide, which mediate the fusion of viral and cellular membranes after conformational rearrangements (5, 10). It binds to ACE2 and mediates membrane fusion during viral entry (5, 9, 10). After the spike protein binds to ACE2, TMPRSS2, a host cell molecule, cleaves the spike protein and generates a range of hydrophobic amino acids that quickly degradates itself (8, 10). Mutations emerged in the RBD can increase viral replication, making the virus more contagious and enable the virus to evade vaccine-elicited neutralizing antibodies (10, 11).

The replication-dependent RNA polymerase in most RNA viruses does not exhibit a proofreading activity. However, coronaviruses express a 3′-to-5′ exoribonuclease in non-structurel protein 14 (nsp14-ExoN) that is main enzyme in RNA virus replication. All molecular studies have demonstrated that nsp-14-ExoN exhibits an RNA proofreading function that can partially correct mutation emerging during virus replication (13). Although coronaviruses contain a genetic proofreading mechanism to continue their RNA genomes, mutations constantly occur in the viruses, with approximately 9.8 × 10^–4^ substitution/site yearly (14, 15). As other viruses, SARS-CoV-2 adapts to novel environment through constantly emerging mutations generated by natural selection (10). Because the spike protein is a key player in binding to ACE2 during viral entry, the mutations emerged in the spike protein can make the SARS-CoV-2 more transmissible and more infectious and modulate tissue tropism and the clinical outcome (8–10, 16). For instance, the viruses carrying D614G mutation, identified by Korber et al. (17) have been demonstrated to be more contagious, spreading worldwide during 3 months. Although most mutations in the SARS-CoV-2 genome are considered to be either mildly deleterious or relatively neutral, a small number of mutations can affect virus phenotype that confers the virus a fitness advantage, leading to alterations in virus biology such as infectivity, transmissibility and antigenicity (8, 10, 16).

In late 2020 and throughout 2021, SARS-CoV-2 generated several new variants with spike mutations that affect the characteristics of the virus, including B.1.1.7 (Alpha), B.1.351 (Beta), P.1 (Gamma), B.1.617.2 (Delta), B.1.1.529 (Omicron) (8–10, 16, 18–20). SARS-CoV-2 B.1.1.7 variant, which is also known as 501.Y.V1 in the GR clade, first emerged in September 2020 in Southest England and has rapidly become the dominant variant in the United Kingdom (10, 21). The Alpha variant contains eight mutations in the spike protein. In addition, Alpha variant has two deletions in the spike protein, one of which is located in an antibody supersite epitope (Y144) (18, 21). Although the other deletion in spike protein increases infectivity, it has a weaken impact on immune evasion (10, 20–24). The N501Y mutation in the RBD may increase binding affinity to the ACE2 (25). Epidemiological studies have demonstrated that the Alpha variant has spread about 50% faster than previously identified variants in the United Kingdom (26), so far, the strain has spread to more than 160 countries (6, 10, 25, 26). In February 2021, researchers have identified B.1.1.7 lineage with E484K mutation to be new VOC in the United Kingdom and then United States (6, 25). The sensitivity of the Alpha variant containing K484E mutation to immune sera from vaccinated individuals with the Pfizer/BioNTech has been found to be sixfold decreased (25, 27).

The Beta (B.1.351) variant, also known as 501Y.V2, has first been detectd in late 2020 in Eastern Cape, South Africa and has since become dominant locally (21, 22, 25). The Beta variant has three RBD mutations, including K417N, E484K and N501Y and five NTD mutations, including a deletion within the NTD supersite at positions 242–244 (10, 25). Epidemiological studies suggest that the Beta variant was found to be about 50% more transmissible than previously reported variants (22, 23). The Beta variant has been associated with reduced sensitivity to many monoclonal antibodies (mAbs), and significant immune evasion after natural infection and vaccination (28). The Gamma (P.1) variant emerged in Brazil in December 2020, which contains ten mutations in the spike protein (7, 23, 25). The Gamma variant has three RBD mutations, including N501Y, E484K, and K417T and five NTD mutations. NTD L18F mutation was demonstrated to prevent the binding of NTD-targeting neutralizing antibodies (28). Because many of these mutations are located in the antigenic supersite in the NTD or in the RBM, the mutations can affect the efficacy of existing monoclonal antibody therapies or vaccines (21). The SARS-CoV-2 variant with K417N, E484K and N501Y substitutions that affect key sites in the RBD may have functional importance (23). The variants containing the combination of N501Y, E484K, and K417N/T exhibit considerable decreased sensitivity to immune response induced by vaccines and convalescent sera (25). All studies suggest that D614G, B.1.1.7, B.351 and P.1 variants are more transmissible and cause more severe disease than original Wuhan SARS-CoV-2 lineages (17, 21, 26, 29).

The B.1.617 variant first emerged in the state of Maharastra in India in late 2020 (20, 30–33). In a few weeks, the B.1.617 variant has become the dominant lineage across India and has spread to more than 60 countries, including the United States, Singapore and the United Kingdom (20, 32). The B.1.617 variant contains three main subtypes, known as B.1.617.1 (the original B.1.617), B.1.617.2 and B.1.617.3 carrying diverse spike mutations in the NTD and the RBD which may increase their immune evasion potential (20, 33). The first two subtypes were identified in December 2020 and the third was detected in February 2021 in India (6, 20). Delta (B.1.617.2) variant accounts for 77% of viruses circulating in United Kingdom between June 2 and 9, 2021 (20). The World Health Organization (WHO) designated B.1.617.2 strain as variant of concern (VOC) (32). Delta variant spreads about 60% faster than the alpha variant (6, 20, 33). The Kappa (B.1.617.1) and the Delta variant harbors mutations in various regions of the SARS-CoV-2 genome, such as the RBD mutation L452R, S1-S2 cleavage site mutation P681R and mutations within orf3, orf7a and the nucleocapsid gene. While the Kappa variant has the RBD mutation E484Q, the Delta variant contains the RBD mutation T478K (28, 33). The strain with E484Q can evade immune responses induced by vaccine or convalescent sera. The Delta variant has 8 mutations in the spike protein, including T19R, D157–158, L452R, T478K, D614G, P681R, and D950N (23, 31). Several of these mutations may affect immune response to the key antigenic regions of the RBD and deletion of the NTD (34). The strain with P681R mutation may have increased replication ability, which causes higher viral load and enhanced transmission (35). The 452R and 478K mutations of delta variant may also increase transmissibility (25, 33). The B.1.617 variants can evade the immune response triggered by vaccine, or by convalescent sera (25, 33). The 452R and 478K mutations may play a role in evading of the virus from immune responses (20, 27). The researchers suggested that B.1.617.1 variant carrying E484Q has been observed to be more associated with vaccine escape (20, 27). This mutation is not found in Delta variant (27). The subtypes of the B.1.617 lineage have decreased sensitivity to some monoclonal and polyclonal antibodies (33).

In early November 2021, the B.1.1.529 variant has first been identified in Botswana and South Africa (18, 19). Since then, the variant has rapidly become dominant variant in South Africa and dozens of countries worldwide have reported Omicron cases. On November 26, the WHO designed the strain as a VoC and named it as Omicron (18, 19). The Omicron variant contains a larger number of mutations in the spike protein, about 32 mutations, several of which (such as 69–70 del, K417N, T478K, N501Y, and P681R) are shared with the other VOCs, including the Alpha, Beta, Gamma, and Delta variants (18, 19). Additionally, the Omicron variant harbors three deletions and one insertion in the spike protein (19). These genetic alterations enhance viral binding affinity, increase viral replication and viral load, and induce immune escape (18, 19, 36, 37). Mutations in the RBD of the spike protein have been found to be associated with increased viral replication, viral load, transmissibility and immune evasion after previous infection and vaccination (19). Mutations near the furin cleavage site are expected to increase transmissibility (38). Both N501Y and D614G mutations increase viral replication, making the virus more contagious (18, 19). So far, collected data regarding the impact of Omicron variant on clinical presentation are insufficient. However, early reports from the South African clinicians indicate that the rate of hospitalization due to Omicron infections is lower than that for Delta variant-related infections. The South African clinicians also demonstrate that patients with Omicron variant are usually younger people who have clinical symtoms and findings similar to that of previous variants (18, 19). The Omicron variant has a larger number of mutations in the spike protein than previous VoCs and some of the mutations, such as K417N and T478K mutations can confer the virus to avade immune responses triggered by vaccines (18, 19, 36, 37, 39). Given that these features of Omicron variant, Omicron may have an impact on the clinical efficacy of COVID-19 vaccines (18, 19). The Omicron variant contains three subvariants, including BA.1, BA.2, and BA.3, with extremely different genetic landscapes (40, 41). Preliminary studies showed that Omicron BA.2 is remarkably more transmissible than BA.1 subvariant (40, 42). BA.2 subvariant has become the prevalent Omicron subvariant in Denmark, the Philippines, and South Africa in the past few weeks (40, 42). Recently, researchers found BA.2 sublineage to be associated with an increased susceptibility of infection for unvaccinated individuals, vaccinated individuals and booster vaccinated individuals, compared to BA.1 sublineages (42).

## Antigenic Features of the SARS-CoV-2 Spike Glycoprotein

Understanding the functions of the spike glycoprotein and its interaction with the immune system requires information of the structures, conformations and distributions of S trimers within virions (8, 9, 11, 19). The SARS-CoV-2 is an envelope, positive-sense, single-stranded RNA virus which belongs to the betacoronaviridae family, which has largest genome, genome lenght ∼ about 30,000 nucleotide, among single-stranded RNA viruses (5–7, 12, 14). The genome consists of a 5′-untranslated region (UTR), non-structurel genes (ORF1a and ORF1b), which encode polyproteins pp1a and pp1b, structurel genes which encode spike (S), envelope (E), membrane (M), nucleocapside (N) proteins, and several open reading frames (ORFs) that encode accessory proteins, 3′-UTR with poly A tail (5, 6, 43, 44). Polyproteins pp1a and pp1ab are cleaved with autoproteolytic enzyme into 16 non-structural proteins (nsp1–16) that play significant roles in viral replication, transcription, immunomodulation, gene transactivation, and resistance to innate antiviral response (6.44). SARS-CoV-2 genome encodes spike proteins, protruding from the surface of mature virions and provide specificity for cellular entry receptor (45). Envelope protein plays a pivotal role in the pathogenesis of COVID-19 infection (5, 6). The nucleocapsid binds to viral RNA and affects the replication ability of SARS-CoV-2 (5, 6). The M protein has three domains, including C terminal, transmembrane and N terminal-domain, and it is required for the assembly and budding of virions (6). Accessory proteins play an important role in evading the innate immune response by interfering with the synthesis of IFN and blocking critical signaling pathways within the cell (46). NSPs are functional proteins that exhibit significant roles in viral replication and methylation and can promote immune responses to infection (5).

The spike protein plays critical roles in viral infection and pathogenesis of COVID-19 infection (45). The spike is a transmembrane glycoprotein that forms homotrimers protruding from the viral surface. SARS-CoV-2 entry into host cells is mediated by the spike glycoprotein (8, 9, 11, 47). The SARS-CoV-2 spike protein is produced in the rough endoplasmic reticulum of infected cells (45). The spike protein is a glycoprotein which comprises two functional subunits, including S1 and S2 (5, 9, 12). The S1 subunit is composed of 672 amino acids (residues 14–685) and harbors an N-terminal domain (NTD), a RBD, and two subdomains (SD1 and SD2) (5, 45). The RBD specifically engages the host cell ACE2 receptor (5). The SARS-CoV-2 virus uses different domains within the S1 subunit to recognize an entry receptor (9). The S2 subunit contains 588 amino acids (residues 686–1273) and harbors an N-terminal hydrophobic fusion peptide (FP), two heptad repeats (HR1 and HR2), a transmembrane domain (TM), and a cytoplasmic tail (CT) (45). S2 subunit is responsible for fusion the membranes of viruses and host cells (5, 9). The cleavage site at the boundry between the S1 and S2 subunits is called as S1/S2 protease cleavage site (5, 7). Host proteases cleave the spike protein at the S2’ cleavage site to activate the proteins which is critical to fuse the membranes of viruses and host cells through irreversible conformational change (9). The RBD in the spike protein is the most variable part of the coronavirus genome (12). Six RBD amino acids were found to be pivotal for binding to ACE2 receptors and for determining the host range of SARS-CoV-2-like viruses. Five of six residues differ between SARS-CoV-2 and SARS-CoV (5, 12). The spike protein is reqiured to initiate infection (9). It binds to the ACE2 to mediate viral entry. The spike protein also determines tissue and cell tropism (5, 10). Mutations in the spike protein may alter the host range of the virus and enable the virus to cross species barriers (9, 10, 16). Membrane fusion is mediated by the large type I transmembrane spike protein on the viral envelope and the cognate receptor on the surface of host cells (8, 11). Surface location of the spike protein confers it a direct target for host immune responses, making it the main target of neutralizing antibodies (45). Spike glycoprotein is a key target for vaccine, antibodies and diagnostics (47).

The RBD is a key player within the S1 unit. It contains a core structure and receptor binding motif (RBM), which is the most variable part of spike protein that is important for binding to the outer surface of ACE2 (9). The spike protein binds to ACE2 receptor and host proteases such as transmembrane proteases serine 2 (TMPRSS2) promote viral uptake and fusion (5, 7, 9, 45). ACE2 and TMPRSS2 are intensively expressed in airways, lung, nasal/oral mucosa, and the intestine (7, 48). SARS-CoV-2 uses either of two host protease enzymes to break in: TMPRSS2 or cathepsin L (5, 9). SARS-CoV-2 efficiently uses TMPRSS enzyme. Priming of the S glycoprotein by host proteases is another critical proceses modulating tropism and pathogenicity (5, 8, 9). First, TMPRSS2 cuts a site on the S2 subunit (5). This cut generates a range of hydrophobic aminoacids in the spike that quickly hide themselves into the nearest membrain -that of host cell (8, 9). Next, the extended spike folds back onto itself and promotes the viral and cell membranes to fuse (8). The virus then releases its genome into the cell (5, 8). Besides receptor binding, the proteolitic cleavage of coranavirus spike proteins via host-derived proteases is a pivotal process for fusion (5, 8, 9). SARS-CoV was demonsxtrated to use the cell surface serine proteases, such as TMPRSS2 for priming and entry, although the endosomal cysteine proteases cathepsin B (CatB) and CatL, can also assit this process (5). Virus and host membranes fuse after the TMPRSS2 enzymes cuts a SARS-CoV-2 spike protein (5, 8, 9). The SARS-CoV-2 spike glycoprotein contains a furin cleavage site at the boundry between the S1/S2 subunits, which is processed during biogenesis and sets this virus apart from SARS-CoV and SARS-related CoVs (9). The SARS-CoV-2 spike glycoprotein may be thought a conformational machine that mediates viral entry by rearranging from an unliganded stage through prehairpin intermediate state (45).

**FIGURE 1:**
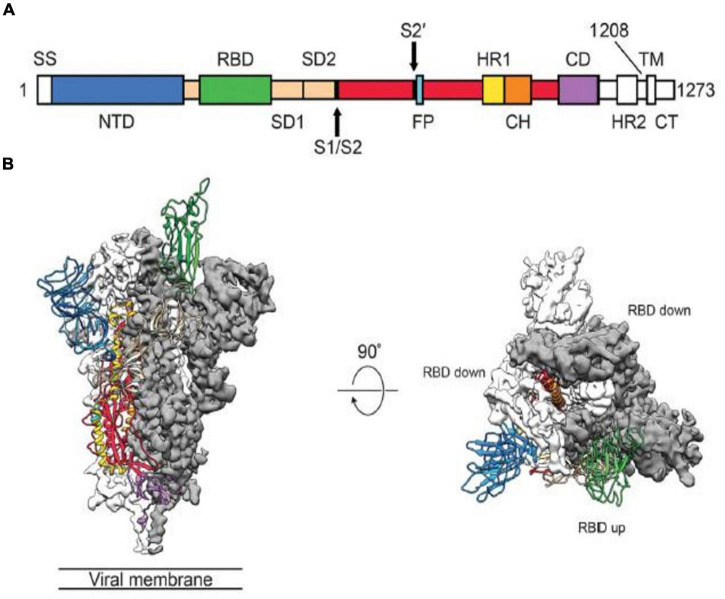
Structure of the SARS-CoV-2 Spike Protein in the Prefusion Conformation. **(A)** Structures of the SARS-CoV-2 Spike Protein. SS, signal sequence; S2’, S2’ protease cleavage site; FP, fusion peptide; HR1, heptad repeat 1; CH, central helix; CD, connector domain; HR2, heptad repead 2; TM, transmembrane domain; CT, cytoplasmic tail; NTD, N-terminal domain. Arrows indicate protease cleavage sites; RBD, receptor binding domain. **(B)** The Prefusion Conformation of the SARS-CoV-2 Spike Protein. To engage a host cell receptor, the RBD undergoes hinge-like conformational movement. Down conformation corresponds to the receptor-inaccessible state and up corresponds to the receptor accessible state. Modified from Wrapp et al. (47).

SARS-CoV-2 uses conformational masking and glycan shielding to hide itself from the immune response (8). SARS-CoV-2 spike protein is surrounded by sugar molecules, which hide it from the host immune system (8, 9). The spike glycoprotein on the virion is a glycosylated trimer, each protomer of which contains 1260 amino acids (42). Each SARS-CoV-2 virion has an outer surface which contains 24–40 randomly located spike proteins (8, 11). SARS-CoV-2 S trimers bind to the ACE2 receptor and mediate entry of virions into the cells. SARS-CoV-2 spike proteins are extremely flexible which can hinge at three points on the stalk (8, 11, 47, 49). That confers the spike proteins to flop around, sway and rotate, making it easier for them to scan the cell surface and for multiple spikes to bind to a human cell (8, 49). Receptor binding impairs the stabization of the prefusion primer and results in shedding of the S1 subunit and transition of the S2 subunit to a postfusion conformation (47, 49). Spike protein undergoes an remarkable structural changes from the prefusion form to the postfusion form (49). Overall structures of both prefusion and postfusion forms are highly conserved among coronaviruses (8, 47, 49). In the prefusion conformation, the RBD sits at the top of abroad, trimeric spike above the fusion core (47, 49). Three copies of the RBD are surrounded by three copies of the NTD which exhibit some mobility (9, 44, 46, 47). In the closed prefusion form, all three copies of the RBD are found to be flat on the spike surface, that largely occlude the receptor binding site (11). However, in the open prefusion form, one or multiple RBDs lift to expose the receptor binding site (Figure 1) (9, 47, 49, 50). The surface of the trimer is extensively glycosylated with 22 potential N-linked glycosylation sites per monomer (9, 47, 49, 51). After receptor binding, structural transition of the prefusion conformation to the postfusion conformation brings the fusion peptid and the transmembrane domain together at one end of a long, needle like structure centered around three-helix bundle (49, 51). Five N-linked glycans are spaced along the length of postfusion spike (49, 52). To engage a host cell receptor, the RBD of S1 undergoes hing-like conformational movement that transiently hide or expose the determinants of receptor binding (8, 9). While “down” conformation is an receptor-inaccessible state, “up” form is the receptor accessible state, which is considered to be less stable (9, 47, 49, 50). Because of the indispensable function of the S glycoprotein, it is a key target for antibody-mediated neutralization, vaccines and diagnostics (47, 49). Explanation of molecular and biological characteristics of the prefusion S structure would confer atomic-level information to guide vaccine design and development (47). Compared with SARS-CoV, SARS-CoV-2 binds to ACE2 an estimated 2–4 times more strongly, because several changes in the RBD stabilize its virus-binding hot spots (8). SARS-CoV-2 variants of concern tend to emerge mutations in the S1 unit of the spike protein, which includes the RBDs and is responsible for binding to the ACE2 receptor. The alpha variant has ten alterations in the spike-protein sequence, which results in RBDs being more likely to stay in the “up” position, helping the virus to enter into the cell more easly (8). The Delta variant contains multiple mutations in the S1 unit, including three in RBD that improve the binding ability of RBD to ACE2 and evade the immune system (8).

Although the RBD is immunodominant, the other spike regions, particularly the NTD play significant roles in antigenicity (10, 49, 53, 54). Researchers have identified four deleted regions (RDRs) within the NTD, modulating NTD antigenicity (10, 54). Structural studies on NTD-specific antibodies 4A8 and 4–8 delineated similar epitop locations toward the upper side the most prominently protruding area the NTD (10). N3 loop is considered to be the most immunogenic regions of the spike protein (10, 25). Six antigenic sites, one of which is recognized by all known NTD-specific neutralizing antibodies and was named the “NTD supersite” have been identified by epitope binning of 41 NTD-specific mAbs (55). Deletions in the NTD were identified repeatedly during the evolution of SARS-CoV-2 and were found to be changing antigenicity (56, 57). The researchers have detected four recurrently deleted regions (RDRs) in the NTD. RDR1, RDR2, and RDR4 are located in NTD loops N2, N3 and N5, whereas RDR3 is found between N4 and N5 in another accessible loop (57). RDR2 and RDR4 deletions can abolish binding of 4A8 (57). RDR2 deletions may play a role in immune escape (10). The 242 base-pair deletion in B.1.351 and H69/V70 and Y144 deletions in B.1.1.7 lineage have been detected. L18F mutation in the NTD has also been identified both in alpha and Beta lineage (25). These NTD mutations decrease sensitivity to neutralizing antibodies. Deletions at H69/V70 do not confer antibody evasion, however the deletion makes SARS-CoV-2 more susceptible to deleterious escape mutation in the RBD, such as Y453F (10, 25).

## Key Spike Mutations Affecting the Biological Functions of SARS-CoV-2

The novel SARS-CoV-2 variants are continuing to emerge globally throughout the COVID-19 pandemic. The RNA-dependent RNA polymerase (RdRp) and recombination can generate the replication errors, causing genetic diversity of SARS-CoV-2. The recombination capacity of coronaviruses depends on the strand switching ability of RdRp, and it may have a relevant role in the evoluation of the virus (10, 13). Coronaviruses emerge mutations at slower speed compare to other RNA viruses because they contain proofreading 3′-to-5′ exoribonuclease (nsp14). However most studies have demonstrated that SARS-CoV-2 accumulates two-single nucleotide mutations per month in its genome (15). Mutations emerged in the spike protein can affect the transmission of the virus, cell tropism, and viral pathogenicty (5, 7, 10, 45). Mutations can also affect neutralization triggered by existing COVID-19 vaccines and diagnostic assays (7, 28, 43). Recent studies have demonstrated that only the variants carrying mutations with relevant biological functions showed high transmissibility (9, 23). These key mutations can influence clinical outcomes of COVID-19 infection, viral transmission and evaiding ability of the virus to neutalizing antibodies elicited by vaccines (9, 23, 25). Fallowing RBD of spike protein binds to ACE2 receptor, the SARS-CoV-2 infects cells (5, 6, 8). Therefore, these key mutations may have an impact on the binding ability to ACE2, for example the N501Y mutation in the spike protein may enhance the binding capacity to ACE2 (23).

In late 2020 and early 2021, variants with mutations affecting the biological functions of the virus, including Alpha (B.1.1.7), Beta (B.1.351) and Gamma (P.1) have been identified (6, 7, 9, 10). Korber and colleagues have identified the earliest spike D614G mutation constituted by adenine (A) to guanine (G) nucleotide mutation at position 23.403 in the original Wuhan reference strain in January 2020, in Germany (17). They showed that SARS-CoV-2 variant with D614G mutation has spread quickly through Europe and North America and following 1 month the variant with D614G muatation became dominant strain worldwide (17). The mutation confers fitness advantage to the authentic Wuhan lineage and increases viral infectivity. Several trials suggested that SARS-CoV-2 variant with the D614G mutation have increased transmissibility (17, 22). Spike protein D614G mutation alters SARS-CoV-2 fitness that enhances viral replication through enhancing the infectivity and stability of virions (17, 22). However, viruses with D614G mutation alone do not exhibit antigenic difference (21). Fallowing the emergence of D614G mutation, the B.1.258 variant with N439K mutation in the RBM emerged and spread in European countries (58). N439K mutation increases the binding affinity for the ACE2 receptor and weakens the immune response triggered by monoclonal and polyclonal antibodies in convalescent sera (58). The B.1.1.298 lineage containing Y453F mutation within the RBM has been identified in Denmark, that enhances ACE2-binding affinity (56). The B.1.1.298 variant also contains D69–70 which is an amino-terminal domain (NTD) deletion (59). D69–70 may alter the conformation and generate NTD loop, increasing infectivity (10, 60).

**FIGURE 2:**
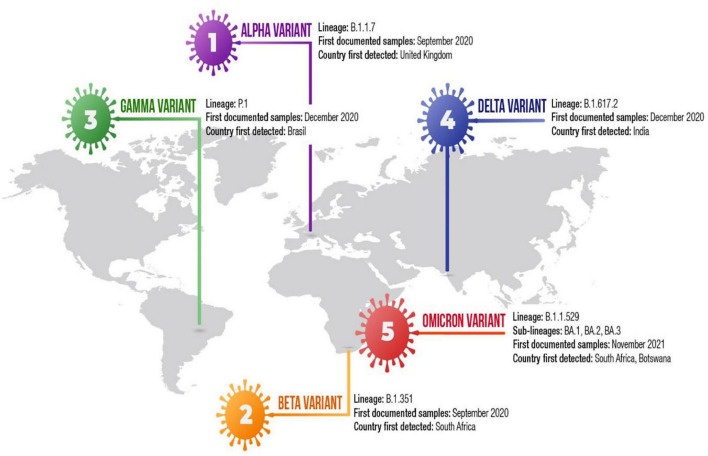
Timeline of the SARS-CoV-2 variant of concern.

So far, five SARS-CoV-2 variants of concern (VOCs), including Alpha (B.1.1.7), Beta (B.1.351), Gamma (P.1), Delta (B.1.617.2) and Omicron (B.1.1.529) variant have been identified (Figure 2) (10, 18, 19, 28). Recently, Alpha variant with E484K in the United Kingdom and the US-Epsilon (B.1.427/29) variants have been reported as VOCs (6, 25). These variants have emerged multiple changes in their genomes, including mutations and deletions in the spike protein (61). The first Alpha variant genomes have been sequenced in the United Kingdom from a sample obtained in October 2020 (10, 25). The Alpha variant has 23 mutations (6, 10, 15). The Alpha variant contains six amino acid mutations in the spike protein, including N501Y, A570D, P681H, T716I, S982A, and D1118H, and two NTD deletions at positions 69–70 and 144 (10, 21, 23, 25). The Alpha variant also contains non-spike mutations including nsp6: D106–108 and the nucleocapsid mutations D3L, R203K, and G204R (25, 52, 62). Phylogenetic analyses have demonstrated that the Alpha variant has been found to be associated with higher growth rate than that of other lineages (25). The Alpha variant was also associated with a higher viral load, particularly in upper-airway (10). Epidemiological studies demontrate that Alpha variant is nearly 50% more transmissible than previously reported United Kingdom lineages. In addition to N501Y that may reduce neutralization by some mAbs, DY144 may exhibit an antigenic effect (10). This deletion may alter the conformation of the N3 NTD loop and was showed to abolish mAbs-mediated neutralization (10, 25). NTD-specific neutralizing antibodies may play dominant role in diminishing neutralization in COVID-19 patients with Alpha variant (10). The D 69–70 prevents the amplification of one of three genomic segments, precluding PCR from giving correct results (59). The Alpha variant is sensitive to immune response mediated by mAbs. The variant rarely weakens immune response to convalescent plasma from previously infected individuals. The combination of DY144 and E484K affect polyclonal antibody response (10, 23). N3 loop, which AY144 changes, is thought to be one of the most immunogenic region of the spike protein and mutations at position 484 weaken neutralization by monoclonal antibodies (10). The Alpha variant contains an N501Y mutation, at the 501st amino-acid position of the spike protein, the amino acid N asparagine is replaced by the amino acid tyrosin. The Alpha N501Y mutation is located within the RBD and may enhance ACE2 receptor affinity (14). P681H mutation in the RBD has biological significance (10, 23). D69–70 in the spike protein was associated with immune evasion (10). In February 2021, the Alpha variant with E484K mutation has been reported as a new VOC (VOC-202102/02) by Public Health England (PHE). The Alpha variant was not reported in the United Kingdom since March 2021, however, sequencing data have demostrated that the variant has been continuing to spread other countries (6). Epidemiological studies showed that the Alpha variant is more contagious than original Wuhan SARS-CoV-2 strains.

SARS-CoV-2 Beta (B.1.351) variant has been identified in late 2020 in Eastern Cape, South Africa (10, 21, 22). The Beta variant contains nine mutations in the spike protein, of which K417N, E484K and N501Y mutations in its RBD have functionally significant (22). This variant has also five mutations in the NTD, including a deletion within the NTD supersite at positions 242–244 (10, 21, 22, 25). NTD deletion, D243–244, breaks binding by the antibody 4A8 and L18F (57). The R246I mutation also emerges within the NTD supersite and may influence antibody binding (10, 54). The combination of K417N, E484K with the NTD mutations which are found in the Beta variant genome can weaken immune response through reducing neutralization induced by RBD-specific and NTD-specific antibodies (10). Many of these mutations emerged in the NTD or in the RBM which is major target of potent virus neutralizing antibodies can affect the effectiveness of current monoclonal antibodies or vaccines (21, 22). Wibmer and colleagues demonstrate that pseudovirus expressing the Beta variant spike protein completely escape three classes of therapeutacillay relevant antibodies (62). Recently, a study using pseudotyped viruses indicated that the Beta variants do not confer an increased infectivity in multiple cells except for murine cells that overexpress ACE2 receptors (63). Chen et al. (64) showed that the Beta variant escapes monoclonal antibody-elicited neutralization.

Both SARS-CoV-2 Alpha and Beta variants have an increased transmissibility and high number of mutations in the spike protein that can cause antigenic alterations that influence immune response to monoclonal antibodies and existing vaccines (23, 25). The E484K mutation interacts with the hotspot of ACE2 and may increase the immunological resistance to neutralization elicited by monoclonal and human serum antibodies (23). Chen and collegues observed that many neutralizing mAbs engaging the RBD or NTD and immune sera triggered by mRNA vaccine demonstrated reduced inhibitory activity against viruses carrying an E484K mutation (64). Greaney and colleagues have also showed that viruses containing an E484K mutation could avade neutralization by polyclonal human serum antibodies (65). Given that existing data, E484K mutation may have altered the antigenic properties of SARS-CoV-2 (23, 65). Therefore, the Beta variant containing E484K mutation can evade immune response (23). Sequencing studies have demonstrated that K417N/T mutation exhibits a weakened impact on binding ability (66). However, MASCp6 mouse models containing both N501Y and K417N mutations have been found to be 100% fatal in aged male mice (67). L452R mutation has been demonstrated to decreases the binding ability of antibodies to spike protein obtaining from convalescent sera (23, 64). Although the Q677 mutation was identified at seven SARS-CoV-2 variants so far, its effect on the infectivity of the variants has not been determined (68).

The Gamma variant (P.1) has first been identified in Japan in early 2021, in travelers from Brazil to Japan (23). The variant contains total 21 mutations, ten of which are located in the spike protein, including L18E, T20N, P26S, D138Y, R190S, K417T, E484K, N501Y, H655Y and T1027I (10). In addition to the RBD mutations, including K417T, E484K and N501Y, the Gamma variant contains some mutations close to the identified antigenic regions of the NTD, such as L18F, that modulates the binding affinity of NTD-targeting neutralizing antibodies (54). The T20N and P26S mutations also emerge in or near the NTD supersite (54). T20N has a potential glycosylation site which can cause glycan shielding of part of the supersite (10). The Alpha, the Beta and the Gamma variants contain N501Y mutations (21, 23). Some studies suggested that the Gamma variant can infect and cause disease in convalescent individuals infected with other variants (55). Epidemiological studies have demonstrated that the Gamma variant has been determined to be nearly 2.4 fold more contagious than precedingly detected variants (69). Preceding infection with non-P1 SARS-CoV-2 confers the protection against P.1 infection compared with non-P.1 lineages (69). So far, P.1 lineage has spread to 64 countries (10). N501Y mutation enhances ACE2 affinity and increases viral replication in human upper airway cells, making the virus more contagious (62). Viruses containing N501Y mutation alone do not have a significant impact on the neutralizing activity triggered by vaccine and convalescent plasma (21). Recently, novel SARS-CoV-2 variants were identified in the United States (23, 67). A new variant, named 20C-US, which contains Q677 and Q173 mutations in the spike protein emerged in the United States in 2020 (70). The Q617H mutation located near the protease cleavage site of the spike protein can influence the stability of the spike protein (23, 70). Researchers have identified a novel variant, named CAL20C, in Southern California (71). The CAL20C strain has five unique mutations, including one in ORF1a:I4205V, one in ORF1b:D1183Y, three in spike protein: S13I, W152C, and L452R (23, 70). The novel strain is responsible for more than 50% of COVID-19 patients in Los Angeles (23). The new SARS-CoV-2 variant, known as B.1.526 was detected by Columbia University (23, 69). The strain is characterized by multiple mutations in the spike protein, including L5F, T95I, D253G, E484K, D614G, and A701V (72, 73). The new variant has rapidly spread and the variant has accounted for more than 20% of COVID-19 cases in New York (72). B.1.525 lineage emerged in the United Kingdom, on December 2020 and became dominant variant in Nigeria (10). The variant has four mutations in the spike protein, including Q52R, E484K, Q677, and F888I, and a deletion mutation, DH69/DV70, similar to Alpha variant (10). B.1.429 variant which has four spike mutations and B.1.427 variant that contains two spike mutations have first been identified in California (6).

The SARS-CoV-2 B.1.617 variant emerged in the state of Maharashtra, India in late 2020/early 2021 (8, 20, 32). The B.1.617 variant has spread rapidly across India and become the dominant strain in a few weeks. To date, the variant has been detected in many countries, such as the United States, Singapore, and the United Kingdom (20, 31, 32). Given that genomic data, before the B.1.617 lineage emerged, the Alpha variant was dominant strain in Delhi and the state of Punjab (20, 31). In the same period, the B.1.618 strain has been dominant strain in West Bengal (31). However, in a few weeks, B.1.617 variant overtaken B.1.618 in West Bengal and became dominant variant in many states (20, 31). The B.1.617 variant comprises three subtypes, including B.1.617.1 (the “original” B.1.617), B.1.617.2, and B.1.617.3, each exhibits slightly difference on genetic basis (20). Both B.1.617.1 and B.1.617.2 variant carry the L452R mutation in the spike protein, P681R mutation in the S1-S2 cleavage site and some mutations in orf3, orf7a and the nucleocapsid gene (6). WHO designed B.1.617.2 a “variant of concern.” The delta variant is characterized by the spike protein mutations, including T19R, D157–158, L452R, T478K, D614G, P681R, and D950G (40). Some of these mutations can influence immune responses to the key antigenic regions of RBD and deletion of part of the NTD (35). The P681R mutation can confer replication fitness to the virus, causing higher viral load and more transmissibility (38). The B.1.617.2 (delta) variant has two mutations E484Q (glutamic acid E substituted by glutamine Q) and L452R leucine L, altered by arginine R) (14). In addition to two mutations, delta also contains a unique mutation, T478K (threonine T replaced by lysine K) (14). Epidemiological studies have demonstrated that the only B.1.617.2 variant is associated with greater public health risk (6). The B.1.617.1 variant has been reclassified to a VOI (Kappa variant) that its global prevalance appears to be declining. The prevalance of B.1.617.3 is low and it is no longer classified as either a VOC or VOI (6). Epidemiological data indicate that the variant is highly transmissible (20, 31).

In early November 2021, the B.1.1.529 lineage has been identified in Gauteng Province, South Africa (18, 19). The variant contains about 30 mutations, 3 deletions and one insertion in the spike protein and some mutations outside of the spike protein (18, 19, 36, 37, 39). Several of the mutations, such as 69–70 del, K417N, T478K, N501Y, and P681R, are shared with the other VOCs, including the Alpha, Beta, Gamma, and Delta variants (18, 19). On 26 November, the WHO designated the B.1.1.529 lineage as a variant of concern and named it as Omicron (18, 19). In a few weeks, the variant has spread quickly and become dominant variant in South Africa. So far, dozens of countries worldwide have reported Omicron variant-related COVID-19 cases. The extremely rapid increase in the number of the Omicron variant-related COVID-19 patients in South Africa indicates that the variant has fitness advantage over Delta variant (19, 74). The Omicron variant seems to be more contagious than other VOCs (19). Although, the data are scarce and incomplete, preliminary reports indicate that the Omicron variant is associated with less severe COVID-19 infection than the infection caused by Delta variant (19, 36, 74, 75). The Omicron variant-related mild COVID-19 infection in South Africa can be related the fact that the country has young population, many of whom have already been exposed to SARS-CoV-2 (19). Epidemiological studies demonstrated that about one-quarter of South Africans are vaccinated with existing COVID-19 vaccines and a large proportion of the population is estimated to have been infected with SARS-CoV-2 in previous waves (19). Mutations in the RBD of the spike protein weaken the ability of neutralizing antibodies to recognize the virus and block infection (18, 19). K417N and T478K mutations can confer the virus to avade immune responses triggered by vaccines (19). Preliminary studies investigating the ability of Omicron variant to evade immune responses indicate that the variant can weaken the potency of neutralizing antibody mediated by vaccination and prior infection (18, 19). Epidemiological studies documented that the Omicron variant has been associated with an increased risk of reinfection (19, 39). However, it is not clear whether the variant can cause more severe diseases than other VOCs (18, 37). Researchers are working intensively to determine potential impact of the Omicron variant on vaccine effectiveness. Preliminary experimental data demonstrate reduced neutralizing antibody response to Omicron variant compared to the Delta variant (76)

**FIGURE 3:**
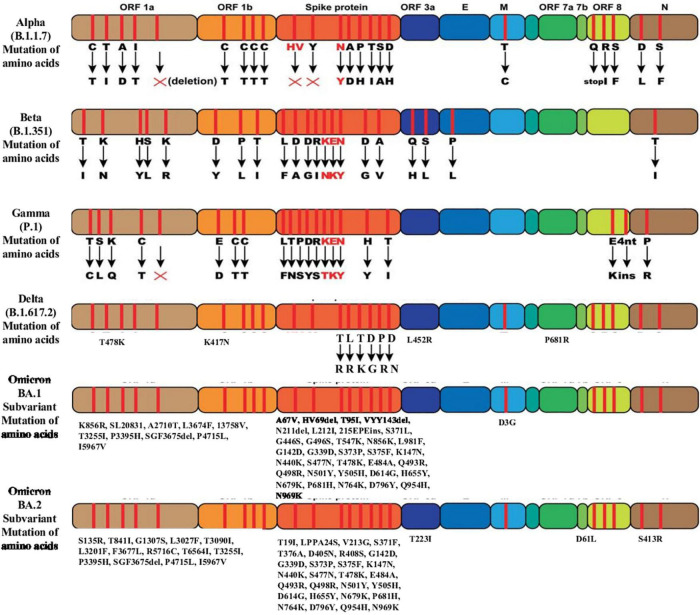
Mutation of amino acids of the SARS-CoV-2 variants of concern.

The Omicron variant has three subvariants, including BA.1, BA.2, and BA.3 sublineages. Virological landscapes of Omicron BA.2 subvariant, such as transmissibility, pathogenicity, and resistance to the vaccine-induced immunity and antiviral drugs differ from BA.1 and BA.3 subvariant (77). Current data suggest that the BA.2 sublineage has a growth advantages over other circulating variants (42). Preliminary studies showed that Omicron BA.2 subvariant spreads faster and substantially more transmissible than BA.1 subvariant (40–42). The Omicron BA.2 subvariant has spread rapidly in countries including Denmark, the Philippines and South Africa in the past few weeks (40). BA.1 and BA.2 differ by approximately 40 mutations, in addition to a key deletion of position 69–70 in spike region of BA.1 compared to BA.2 (40). BA.1 and BA.2 lineages have 51 mutations in their genome, 32 of which are common to both lineage, whereas each lineage has 19 unique mutations (40, 41). Among 32 mutations, 21 are located in the spike protein and the rest 11 mutations are present in the other four coding regions (41). BA.2 sublineage has been found to be associated with an incerased susceptibility of infection for unvaccinated and vaccinated individuals (Figure 3) (42).

## Can SARS-CoV-2 VOCs Blunt Neutralization Triggered by Vaccines?

The remarkably quick development of safe and effective vaccines which limit the burden of COVID-19 infection is a historical success (74). However, fundamental questions regarding the existing vaccines, including the impact of VOCs on vaccine effectiveness, the mechanisms of protection against the COVID-19, the timing between vaccine doses, the effect of vaccines on asymptomatic infection and the duration of vaccine-elicited immunity, remain unanswered (73, 74). The first vaccine development studies have been started in March 2020 and progressed at unprecedented speed throughout 2020 (25, 74–76). Data from several phase III vaccine efficacy studies have been reported at the end of 2020 and have clearly been demonstrated vaccine efficacy (75, 78). These data provided the approval and rollout of these vaccines (75, 78). mRNA vaccines which were developed by Moderna and Pfizer/BioNTech and the viral-vectored AstraZeneca vaccine have been approved (25). To date, WHO has authorized two inactivated vaccines (BBIBP-CorV, CoronaVac), two viral vector vaccines (AZD1222, Ad26COV2-S) and two mRNA vaccines (mRNA1273, BNT162b2) to prevent COVID-19 infection (79). With the succesfully deployment of higly effective vaccines in several countries, researchers and clinicians thought that the global effort in vaccination would control pandemic (74). Unfortunately, the emergence of VOCs temper our initial optimism (25, 74). VOCs have been emerging since the beginning of the Covid-19 pandemic, which are generally more transmissible variants (19). SARS-CoV-2 VOCs can exihibit resistance to the vaccine-elicited immunity (75). Additionally, because some of VOCs have increased transmissibility or virulence, the vaccination programs will become increasingly significant (76). Sequencing studies investigating novel mutations and variants are ongoing intensively. The main goal of these studies is to identify new mutations rapidly and to determine their impacts on viral replication, transmissibility, clinical presentation and effectiveness of the current vaccines (76). Many researche groups are sharing their sequence findings with GISAID (Global Initiative on Sharing All Influenza Data) (76). It is clear that the global efforts against VOCs must be both timely and scientific approaches (25, 76).

Although some vaccines have been approved and rollout succesfully in many countries, individuals who have been vaccinated so far represent a small fraction of the global population (10). It is a great concern that emerging VOCs can evade neutralizing antibodies elicited by previous infection or vaccines through the spike protein mutations. Laboratory neutralization experiments have shown that many of VOCs have reduced sensitivity to vaccine-elicited immunity (25). So far, Alpha variant has been reported to have no significant impact on vaccine efficacy (28). Using an infectious complementary DNA (cDNA) clone of SARS-CoV-2, Xie and colleagues engineered three SARS-CoV-2 viruses containing key spike mutations from the Alpha and the Beta variants and investigated the impact of SARS-CoV-2 spike 69/70 deletion, E484K and N501Y variants on neutralization triggered by BNT162b2 vaccine (80). The researchers also observed that these mutations have weak effects on virus neutralization induced by two BNT162b2 doses (80). In another study, Tregoning et al. investigated SARS-CoV-2 spike pseudovirus generating either the original Wuhan strain or the Alpha variant spike protein with sera of 40 individuals who were vaccinated with BNT162b2 (81). They found that the immune sera has decreased neutralizing activity against the Alpha variant pseudovirus (81). These data show that the Alpha variant does not evade BNT162b2-mediated immune response (81). Wang and colleagues show that the Alpha variant is resistant to neutralizing activity mediated by most of monoclonal antibodies targeting the NTD of the spike protein and is relatively refractory to a few monoclonal antibodies against the RBD. The Alpha variant does not seem to be more resistant to convalescent plasma or sera from vaccinated individuas (21).

N501Y mutation that is detected in Alpha, Beta and Gamma variant genome, does not affect vaccine-elicited and mAbs-induced neutralization (19, 21, 25). However, variants containing E484K mutation, such as Beta and Gamma variant, can evade neutralizing antibodies mediated by vaccines or previous infection (82). The Beta variant has K417N and E484K mutations that significantly affect the mAbs- and convalescent plasma-induced neutralization (74). Wang and colleagues reported that Beta variant is resistant to most of monoclonal antibodies against the RBM of the RBD (21). The researchers revealed that Beta variant is 6.5 fold more resistant than wild-type pseudovirus to neutralization triggered by BTN162b2 vaccine (21). Same findings have been observed in sera from vaccinated individuals with mRNA-1273 (78). Wibmer and colleagues indicate that pseudovirus containing Alpha variant spike protein completely evades three classes of therapeutically significant antibodies (62). This pseudovirus also escapes convalescent plasma-mediated neutralization (62). The E484K mutation reduces sensitivity to neutralization by 100-fold in some individuals (68, 69). Individuals vaccinated with mRNA-1273 or BNT162b2 show reduced neutralization activity against SARS-CoV-2 viruses carrying E484K and N501Y mutations or the triple combination of K417N, E484K and N501Y (63). Although the Gamma variant has a higher number of mutations in the spike protein than other three VOCs, *in vitro* neutralization experiments with pseudotyped virus showed that the neutralizing activity of BNT162b2-mediated antibodies to B.1.1.7-spike virus and P.1-spike virus is nearly equivalant (83). Recently, experiments using pseudo viruses demonstrate that the Beta variant exhibits resistance to mAbs-induced and vaccine-mediated neutralization (64). Several studies investigated the neutralizing activity of pseudoviruses of 501Y.V1, 501Y.V2 and P.1, by using convalescent sera, vaccine-elicited sera (mRNA-1272 and NVX-CoV2373) and monoclonal antibody (63–65). In all studies, the neutralizing activity was found to be decreased (63–65). However, engineered pseudovirus does not contain all biological properties of the original SARS-CoV-2 virus. Jangra and colleagues showed that the spike protein E484K mutation reduces but does not remove neutralizing activity elicited by convalescent and post-vaccination sera (82).

There are conflicting reports on the efficacy of current COVID-19 vaccines against Delta variant. The Delta variant does not contain N501Y and E484K mutations in its RBD that confer the variant to evade neutralizing antibodies (NAbs) (20, 43). Xie et al. conducted a study investigating the effectiveness of existing Covid-19 vaccines against the Delta variant in England (83). In the study, while the effectiveness of two doses BNT162b2 vaccine against Delta variant-associated symptomatic disease has been found to be 88%, this efficacy was detected to be 67% with two doses AZD1222 vaccine (83). An important reduction in neutralizing antibody level was observed for Delta variant compared with Alpha lineage using sera from individuals who have been vaccinated with BTN162b2 (16). Delta variant exhibits higher binding affinity and infectivity (34). The 156–157 deletion and G158R, I452R, T478K mutations of Delta variant may lead to the reduction of antibody neutralization (63). Before the Omicron variant was identified, Delta variant has been considered to be most transmissible variant (57). The neutralization activity of BNT162b2 vaccine-mediated sera has been investigated by using engineered mutant viruses and three variants, including N501Y variant, 69/70 deletions + N501Y + D614G variant and E484K + N501Y + D614G variant, have exhibited slight effect on neutralization of BNT162b2 vaccine-elicited sera (16). Additionally, Wang and co-workers have studied the immunity, including neutralizing antibody titre and memory B cell responses mediated by mRNA vaccines (mRNA-1273 or BNT162b2 vaccines) in 20 individuals (84). The neutralizing activity of vaccine-elicited sera against pseudoviruses carrying E484K, N501Y, and K417N/E484K/N501Y cluster has been found to be decreased (85).

Frieman et al. from PHE published a non-randomized trial investigating the effecacy of the BNT162b2 and ChAdOx1 vaccines against alpha and delta variants (46). The researchers have used a test-negative design to determine vaccine effectiveness in PHE study (46). The study showed that vaccine effectiveness after one dose was lower by about 12–19% points against delta variant than against alpha variant (46). Vaccine effectiveness after two doses of the BNT162b2 vaccine has been determined to be 94% against the Alpha variant and 88% against the delta variant (43). The corresponding percentages with the ChAdOx1 nCoV-19 vaccine were determined to be 74 and 67% (43).

Abu-Raddad and colleagues have investigated the effectiveness of the BNT162b2 vaccine against the B.1.17 and B.1.351 variant (86). The researchers have demonstrated that the BNT162b2 vaccine was effective against both the B.1.1.7 and B.1.351 lineage-related infection and disease (87). However, vaccine effectiveness against the B.1.351 has been found to be lower than the effectiveness reported by prevous studies (84, 88). The effectiveness against the Beta variant – related Covid-19 infection has been found to be 75.0% (95% CI, 70.5–78.9) (87). Vaccine effectiveness against severe, critical, or fatal disease caused by any SARS-CoV-2 variant has been detected to be 97.4% (95% CI, 92.2–99.5) (81). This finding was consistent with previously reported clinical trial finding (86, 89). The number of patients and follow-up periods are not sufficient to determine vaccine effectiveness against severe disease. Recently, Yadav and colleagues from India have reported that immune sera triggered by BBV152 (Covaxin) vaccination and previous infection have been able to neutralize B.1.617 sublineages (90).

Zhou and colleagues have published a study searching a structure-function analysis of the Beta variant using a serum samples from individuals who received vaccine (61). The researchers have demonstrated that mutations in the RBD enhance ACE2 binding affinity and confer the virus to evade monoclonal antibody-mediated neutralization (61). The Oxford-AstraZeneca- and Pfizer vaccines-elicited antibodies to the Beta variant has been found to be reduced by 9 and 7.6-fold, respectively (61). Novavax vaccine demonstrated 95.6% efficacy against previous SARS-CoV-2 strains and 85.6% against B.1.1.7 variant, However, Novavax showed decreased effectiveness of 60% in South Africa. Wang and colleagues demonstrated that B.1.1.7 is resistant to the NTD mAbs-induced neutralization and relativelly refractory to a few mAbs targeting the RBD (21). The researchers suggested that Alpha variant is not more refractory to convalescent and vaccine sera (21). The key findings of the study were (a) The Beta variant has been detected to be refractory the most mAbs targeting NTD-induced neutralization (b) the Beta variant was also found to be resistant to multible individual mAbs targeting the RBM-induced neutralizaiton (21). Additionaly, the Beta variant was remarkable more resistant to immune response to convalescent plasma (9.4 fold) and vaccinee sera (10.3–12.4 fold) (21). Recently, Planas and colleagues have examined Delta lineage sensitivity to mAbs and to antibodies in sera from Covid-19 convalescent individuals or vaccinated persons (34). Sera from individuals who have received one dose of BioNTech/Pfizer or AstraZeneca vaccines showed minimal inhibition of Delta variant (34). Serum samples collected after first dose of BNT162b2 and AZD1222 vaccines did not significantly neutralize Alpha, Beta, and Delta variants. After the second dose of BNT162b2 and AZD1222 vaccines, sera neutralized 94 and 95% of the Delta variant, respectively. The researchers suggest that Delta variant evades neutralizing antibodies triggered by vaccines or previous infection (34). Omicron variant contains a larger number of the mutations in the spike protein than prior variants and the potential impact of these mutations on effectiveness of existing vaccines is not clear (18, 19). The epidemiological studies searching the impact of Omicron variant on the efficacy of existing COVID-19 vaccines has been ongoing intensively. Preliminary studies documented the Omicron variant blunts the potency of neutralizing antibodies triggered by prior infection and vaccination (19). The variant has some capacity to evade immunity. The Omicron mutations affect immune system less than antibody responses (19, 36, 37). Preliminary laboratory data have documented substantially declined neutralizing activity to Omicron compared to the authontic Wuhan virus or the Delta variant in vaccinated individuals. Neutralizing antibody was detected to correlate with protection against reinfection and vaccine effectiveness against infection (91, 92). Andrews and colleagues have documented that vaccine effectiveness against symptomatic COVID-19 infection caused by the Omicron variant is substantially lower than with the Delta variant (93). The researchers documented that two doses vaccination with BNT162b2 or ChAdOx1 do not provide suffecient neutralizing antibody levels to infection and mild disease with the Omicron variant (93, 94). However, booster vaccination, with BNT162b2 confers a substantial protection against mild disease, and can provide a stronger protection against severe and fatal disease. These data are consistent with preliminary neutralization levels for the Omicron variant published by South African and Germany studies (76, 78). Studies investigating antigenic characterization of the Omicron BA.1 and BA.2 sublineages indicated that polyclonal sera obtained from patients with COVID-19 infection or vaccinated individuals demonstrated a significant loss in neutralizing activity to BA.1 and BA.2 (77, 95–98).

## Conclusion

SARS-CoV-2 is evolving, emerging novel variants with spike protein mutations. In this setting, we have to expect the emergence of novel SARS-CoV-2 variants. So far, five VOCs have been identified, including the Alpha, the Beta, the Gamma, the Delta and the Omicron variant, that have a high number of mutations in their spike protein. Some mutations emerged in the spike protein can confer the virus a fitness advantage that increases viral replication and viral load, making the virus more infectious and more contagious. Epidemiological studies have demonstrated that Delta variant has spread about 60% faster than Alpha variant. the Omicron variant has fitness advantage over the Delta variant and it is more transmissible than Delta variant. Some of the spike protein mutations, particularly mutations emerged in the RBD, can blunt the potency of neutralizing antibodies triggered by existing vaccines and prior infection. Additionally, these mutations confer the VOCs the ability to evade immunity mediated by vaccines. Preliminary laboratory experiments demonstrate substantially declined neutralizing activity to the Omicron variant compared to the authentic Wuhan virus or the Delta variant in vaccinated people. However, booster doses enhance neutralizing antibody response to the Omicron variant. Neutralization elicited by two BNT162b2 or ChAdOx1 doses confers mitigated protection against symptomatic disease with the Omicron variant.

## Author Contributions

The author confirms being the sole contributor of this work and has approved it for publication.

## Conflict of Interest

The author declares that the research was conducted in the absence of any commercial or financial relationships that could be construed as a potential conflict of interest.

## Publisher’s Note

All claims expressed in this article are solely those of the authors and do not necessarily represent those of their affiliated organizations, or those of the publisher, the editors and the reviewers. Any product that may be evaluated in this article, or claim that may be made by its manufacturer, is not guaranteed or endorsed by the publisher.

## Abbreviations

SARS-CoV-2: severe acute respiratory syndrome 2
COVID-19: Coronavirus disease 2019
SARS-CoV: severe acute respiratory syndrome coronavirus
MERS-CoV: Middle-East respiratory syndrome coronavirus
ACE2: Angiotensin-converting enzyme 2
TMPRSS2: Transmembrane proteases serine 2
RBD: Receptor-binding domain
CatB: Catapsin B
NTD: N-terminal domain
VOCs: variants of concern
UK: United Kingdom
US: United States
WHO: World Health Organization
GISAID: Global Initiative on Sharing All Influenza
PHB: Public Health England
BNT162b2 vaccine: Pfizer/BioNTech
AZD1222 vaccine: AstraZeneca
mAbs: monoclonal antibodies.
